# The combined effect of Bassa 50EC and Vitashield 40EC on the brain acetylcholinesterase activity in climbing perch (*Anabas testudineus*)

**DOI:** 10.1007/s11356-018-2112-1

**Published:** 2018-04-30

**Authors:** Nguyen Thanh Tam, Håkan Berg, Nguyen Van Cong

**Affiliations:** 1 Faculty of Fishery, Nong Lam University, Block 6, Linh Trung Ward, Thu Duc District, HCM city, Vietnam; 2 Department of Physical Geography, Stockholm University, SE-106 91 Stockholm, Sweden; 3 College of Environment and Natural Resources, Can Tho University, 3/2 Street, Can Tho city, Vietnam

**Keywords:** Acetylcholinesterase, Chlorpyrifos ethyl, Fenobucarb, Combined effect, Climbing perch, Mekong Delta

## Abstract

The combined effect of Vitashield 40EC (chlorpyrifos ethyl-CPF) and Bassa 50EC (fenobucarb-F) was compared with the effects from exposure to the two pesticides separately, by measuring the brain acetylcholinesterase (AChE) activity in climbing perch fingerlings (*Anabas testudineus*). The experiment was conducted under controlled laboratory conditions and included three treatments containing 0.173 mg/L of CPF, 1.137 mg/L of F, 0.173 mg/L of CPF + 1.137 mg/L of F (M), and a control. The inhibition of the brain AChE activity in fish exposed to F was weaker and shorter than in fish exposed to CPF. The inhibition by the mixture of CPF and F was significantly lower and less prolonged than the inhibition by only CPF but significantly higher than the inhibition by only F.

## Introduction

The Mekong Delta covers an area of about 40 thousand square kilometers accounting for approximately 12.2% of the total Vietnam area, and provides more than 50% of the total rice production of the country (Huy 2011; Berg et al. 2016). During the last three decades, the farming areas and rice production in the delta have increased rapidly, to satisfy the food demand of the increasing national population and to generate export earnings. The total rice cropping area in the Mekong Delta increased from approximately 1.2 million hectares in 1995 to approximately 1.7 million hectares in 2013, while the rice yield increased from 4.0 to 5.8 ton/ha/crop during the same period of time (GSO 2014). In Vietnam, the rice production increased from 26.4 million tons in 1996 to 44.1 million tons in 2013 (GSO 2014). Strategies for increased rice production have mainly relied on intensified farming methods, supported by high-yield rice varieties and increased use of agrochemicals, especially pesticides (Huan et al. 2008; Phong et al. 2010). Hoi et al. (2016) indicated that the pesticides imported to Vietnam have increased from 100 tons per year in the 1950s to the highest of approximately 150,000 tons in 2008 and then dropped to 103,500 tons in 2012. The number of active ingredients has increased from 294 in 2002 to 1085 in 2011 (MARD 2011).

Although the increased use of pesticides has helped to increase the rice yields, it has also been followed by an increased risk for negative effects on the environment and on people’s health (Berg et al. 2012; Sebesvari et al. 2012). Dasgupta et al. (2007), Ky and Ngoc (1998), and Thuy et al. (2003) indicated that the use of pesticides in the Mekong Delta can cause adverse impacts on human health, including skin and eye irritation, headache, dizziness, and shortness of breath. Several other studies also found negative impacts of pesticides on human health in the North of Vietnam (Ky and Ngoc 1998; Murphy et al. 2002; Phung et al. 2012a, b, 2013). Many farmers have three crops per year and often use several different pesticides per crop. Pesticides are often applied in combination, to reduce the working time in the field and with the expectation to prevent several crop diseases at the same time. The combination of different pesticides could potentially result in “cocktail” effects on aquatic organisms in the rice fields. The combined toxic effects may differ from the effect of the separate pesticides, and could be additive or non-additive. In addition, pesticides are spread to other areas, through wind drift and irrigation channels, exposing aquatic organisms to a variety of different pesticides. Although the water concentrations of many sprayed pesticides are not likely to cause any acute toxic effects on aquatic organisms, they may still generate long-term lethal and sub-lethal effects such as reduced growth rate, feeding capability, and swimming activity (Stadlinger et al. 2015).

Anh et al. (2003) indicate that wild fish resources, such as climbing perch and snakehead fish in rice fields and floodplains of the Mekong, have declined by 70% compared to 30 years ago. One of the main causes of this decline is the high use of pesticides (Anh et al. 2003). However, official information about the negative side effects of pesticides on aquatic organisms in the rice fields from the Mekong Delta is scarce.

This study therefore aims to assess how some pesticides commonly used on rice affects fish in the Mekong Delta. Organophosphates (OPs), such as chlorpyrifos ethyl (CPF), and carbamates (CMs), such as fenobucarb (F), are popular insecticides used by many rice farmers. According to the list of registered pesticides in 2013, approximately 158 and 40 different pesticides contain CPF and F, respectively, as the main active ingredients, and are used to control a wide range of pests in the rice fields (Heong et al. 1998; Huan et al. 1999; Phong et al. 2010; MARD 2013). These pesticides are often detected in fresh water systems in the Mekong Delta (Chen et al. 2014). Toan et al. (2013) found fenobucarb to be the most frequently detected insecticides (found in 91% of the samples) in rice field water in the Mekong Delta, with a median concentration of 0.11 μg L^−1^. The water concentration can be much higher directly after the pesticides are applied to the rice field, but decrease quickly within a couple of days (Stadlinger et al. 2016; Tam et al. 2015, 2016a, b). Tam et al. (2015, 2016a, b) found that the concentrations of CPF in rice field water sprayed with 0.64 kg CPF/ha varied between 4.23 and 5.35 μg/L after 1 h and 0.47 and 0.73 μg/L after 1 day, respectively. Tam et al. (2016a) also found that the concentrations of F in rice field water sprayed with 1.5 kg F/ha were 135 μg/L after 1 h and 36 μg/L after 1 day.

The concentration of CPF and F in water is influenced by the amount of insecticides sprayed and on the water volume in the rice field (Tam et al. 2015, 2016a, b; Toan et al. 2013). Tam et al. (2015, 2016a, b) found that the highest water concentrations were found among the rice plants in the fields that received the highest application doses, while the lowest water concentrations were found in the canal of the fields. This was probably because the insecticides were sprayed directly on the rice plants and not on the canal. The larger water volume of the canals probably also diluted the applied insecticides (Tam 2016). Toan et al. (2013) proposed that the slightly higher pesticide levels found during the dry season could be due to the lower water volume during this period of time.

Both CPF and F are known to be highly toxic to freshwater fish and aquatic invertebrates, because of their abilities to inhibit the acetylcholinesterase (AChE) enzyme and to cause malformation of the nervous system (O’Brien 1976; Peakall 1992; Taylor and Brown 1999; Tilak et al. 2001). Gruber and Munn (1998) indicated that, although OP and CM compounds can break down rapidly in the environment, non-target organisms may suffer from chronic effects, including AChE inhibition, for several months in contaminated areas, such as intensive rice farming, where pesticides are applied continuously. According to Peakall (1992), inhibition of AChE results in abnormal respiration, swimming, feeding, and social interactions in aquatic organisms, due to loss of coordination, tremors, muscle spasms, convulsions, and even death.

AChE activity in fish has been used as a biomarker for OP and CM environmental contaminations for many years (Assis et al. 2011; Lionetto et al. 2013). The successful application of this biomarker for monitoring of human health also suggests that it can be used in overall risk assessments of OP and CM compounds, addressing both environmental and health risks. In addition, Lionetto et al. (2013) revealed several advantages of using AChE as a biomarker: the response is easy to measure, it shows a dose-dependent behavior to pollutant exposure, it is sensitive, and it exhibits a link to health effects. However, using AChE as a biomarker to indicate the degree of sub-lethal effects from OPs and CMs on aquatic organisms in natural environments requires baseline information on the normal AChE activity levels in unexposed organisms, which may be very difficult to find in the field. Moreover, the effect on the AChE activity depends on a number of factors such as species, age, and sex.

To monitor the negative side effects of insecticides on fish in the Mekong Delta, it was seen as important to select an indigenous species that is commonly found in the whole delta. Rice fields are natural and important habitats for climbing perch *(Anabas testudineus)*, and this fish species is directly exposed to pesticide levels that can cause negative impacts during all its life stages (Tam et al. 2015a; Stadlinger et al. 2016). Although there are no official reports about the status of climbing perch populations in the Mekong Delta, the perception among local farmers is that the amount of wild fish in rice fields has decreased during the last 25 years (Edwards et al. 1997; Anh et al. 2003; Klemick and Lichtenberg 2008), which could be due to the increased use of pesticides in the delta. Climbing perch is a supplementary source of food and income for many local households, and the declining yield of this species affects the local people’s livelihood and well-being. Climbing perch has been shown to be an important predator of rice pests, such as brown planthopper (*Nilaparvata lugens* (Stål)), and could potentially help to reduce the use of insecticides to control these pests (Nam 2011). Choudhury et al. (1993) found that non-lethal levels of metacid-50 (0.106 ppb) and carbaryl (1.66 ppm) insecticides significantly reduced the gonadosomatic index and the profiles of estradiol-17β in the plasma and ovary of adult, female climbing perch. Jilna and John (2011) found that the exposure to sub-lethal concentration of methyl parathion (2.7 mg/L) not only influenced behavioral activities but also significantly reduced the level of protein content in the fore brain of climbing perch. Binoy et al. (2004) carried out an experiment to investigate the impact of dicofol on the behavior of climbing perch including respiratory rate, swimming performance, feeding behavior, and learning ability. The negative impacts of diazinon on climbing perch using muscle cholinesterase were studied by Cong and Linh (2010). Thus, there are several studies on the effects of pesticides on climbing perch, but information regarding the effects of CPF and F are scarce. The main purpose of this study was, thus, to investigate the negative impacts of Vitashield 40EC (CPF) and Bassa 50EC (F) applied alone and in a mixture on the brain activity of acetylcholinesterase in climbing perch.

## Materials and methods

### Test animals

The tested fish (3–4 g) used in the experiment was purchased from a fish farm in the Co Do district outside the Can Tho city in Vietnam. The fish fingerlings were stored in a 600 L fiberglass tank for 3 weeks to adjust to the experimental conditions. During the acclimatization, the fish were fed daily with *Tubifex sp*. at approximately 5% of total body wet weight. The uneaten feed and feces were removed from the tank 4 h after feeding. Fifty percent of the water volume was replaced, when cleaning the tank. The feeding was stopped 1 day before the start of the experiment.

### Insecticides

An organophosphate insecticide, Vitashield 40EC (containing 40% chlorpyrifos ethyl (≈ 1.141 mol/L) and inert ingredients including emulsifiers and solvents), was bought from the Thanh Son Hoa Nong Company in Vietnam.

A carbamate insecticide, Bassa 50EC (containing 50% fenobucarb (≈ 2.412 mol/L) and inert ingredients including emulsifiers and solvents), was bought from the An Giang Plant Protection and Services Company in Vietnam.

### Experiment design

The experiment was carried out in fiberglass tanks (60 L) containing 40 L of tap water. The water (pH 6 5–7 0) had been aerated for 12 h to remove any residues of chlorine. The experiment included three treatments containing 0,173 mg/l (0.493 μmoles/L) of CPF, 1.137 mg/L (5.486 μmoles/L) of F, and 0.173 mg/L of CPF + 1.137 mg/L of F (M) and a control. All concentrations, including the control, were done with three replicates. The used concentrations of the pesticides corresponded to 10% of the 96-h LC 50 values of CPF (Tam et al. 2015b) and F (Lan 2004) for climbing perch fingerlings.

The stock solutions of CPF and F were achieved by diluting the commercial products 500 times with distilled water. The nominal concentrations of the stock solutions of CPF and F were 800 and 1000 mg/L, respectively, which were equivalent to 2000 mg/L of Vitashield 40EC and 2000 mg/L of Bassa 50EC. The final measured concentrations of the stock solutions were determined following a procedure modified by Laabs et al. (2002) to 760 mg/L of CPF and 950 mg/L of F. The solutions were then added to the three treatments to obtain the different concentrations.

Twenty fish (average weight 7.42 ± 0.15 g) were removed randomly from the stocking tank and added to the experimental tanks, within 5 min after preparing the treatment concentrations, There were no water exchange, aeration, or feeding during the experiment. Dissolved oxygen, pH, and temperature were measured daily at 7:00 am and 2:00 pm using a DO meter (HANNA HI9146) and a pH meter (HANNA HI8314). At day 1, 3, 5, and 7, two fish were randomly caught from each tank, killed, and processed for analysis of AChE activity.

The findings from this study were compared with the results from a similar study exposing climbing perch fingerlings to a mixture of 0.32 kg CPF/ha and 0.75 kg F/ha under field conditions in rice fields in the Mekong Delta (Tam et al. 2016b).

### Cholinesterase assay

The fish was put on ice and the brain was removed and placed in an Eppendorf tube on ice to measure the weight. A glass homogenizer was used to homogenize the brain in 6 mL of 0.1 M phosphate buffer (pH 7.4). The homogenates were transferred into 1.5 mL Eppendorf tube and centrifuged at 268×*g* at 4 °C for 20 min. The supernatant was removed to an Eppendorf tube and kept on ice. The AChE activity was determined within 12 h following the method by Ellman et al. (1961). A cuvette was prepared containing 2.65 mL of 0.1 M phosphate buffer (pH 7.4), 100 μL of 3 mM 5,5′ 2-nitrobenzoic acid. 50 μL of 10 mM acetylthiocholine iodide and 200 μL of the supernatant were added just before the measurement. Blanks contained 200 μL of buffer instead of the supernatant. The AChE activity was measured using a spectrophotometer (Hitachi U2800, Japan) for 200 s at a wavelength of 412 nm. During this time, the increase in absorbance with time was linear. The results of these measurements were expressed as a rate (absorbance per minute), from which the AChE activity was calculated.

### Data analysis

The Kolmogorov-Smirnov test and Levene test were used to check the normality and variance of homogeneity of the data. Data was analyzed with one-way ANOVA and Dunnett’s post hoc test for multiple comparisons using SPSS software for Windows (Ver 17.0; SPSS, Chicago, IL, USA).

## Results

**Fig. 1:**
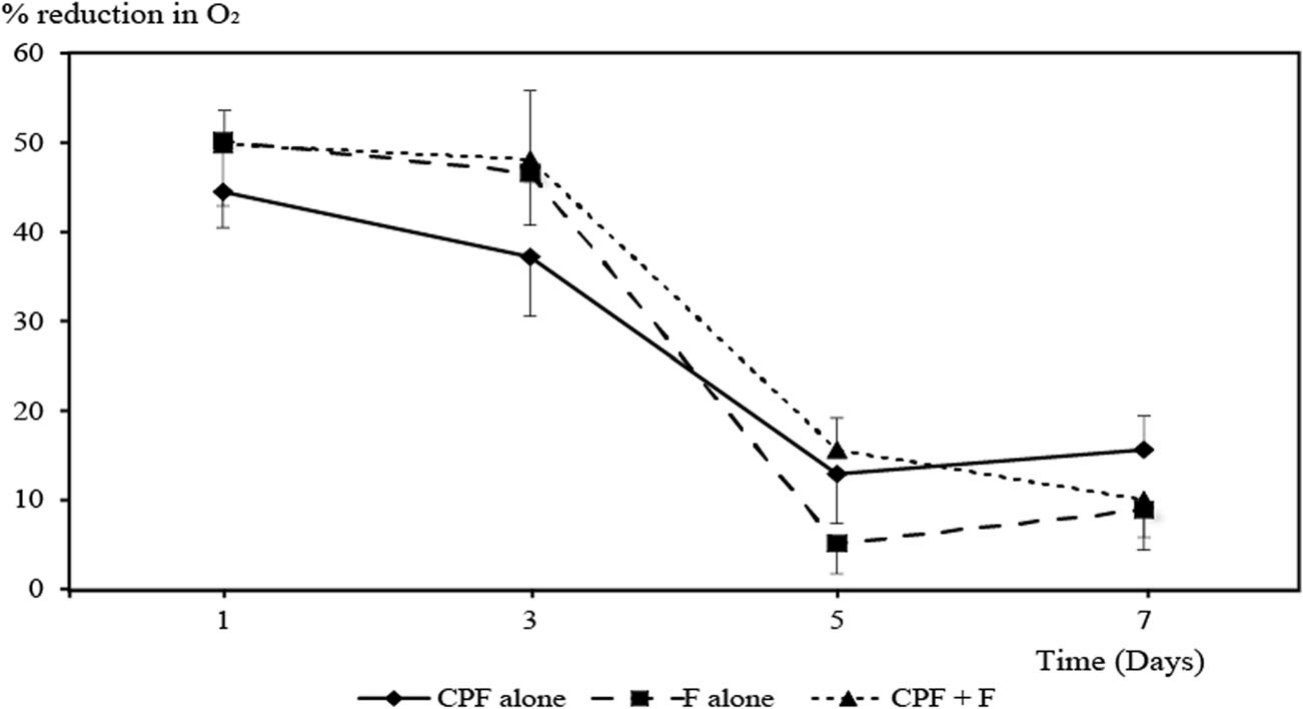
Reduction (%) in dissolved oxygen in the water from the treatments compared to the control after exposure to chlorpyrifos ethyl (♦-CPF alone), fenobucarb (■-F alone), and the mixture (▲-CPF + F). The vertical bars show the standard deviation

The water temperature varied between 23.3 and 23.8 °C, and was not significantly different between treatments at any sampling time (*P* > 0.05). The pH at day 5 ranged between 7.00 and 7.12, which was significantly higher than the pH during days 1, 3, and 7 (from 6.40 to 6.85) (*P* < 0.05). However, there was no significant difference among treatments at the same sampling time (*P* > 0.05). The DO levels in the control at days 1 and 3 were significantly higher than the DO levels in the treatments (*P* < 0.05), but there were no significant differences in DO levels among the different treatments and the control at days 5 and 7 (*P* > 0.05) (Fig. 1). The DO levels during days 5 and 7 in all treatments were significantly higher than the DO levels at days 1 and 3, which indicate a reduced respiration and stress of the fish exposed to CPF and F towards the end of the experiment. Although not statistically significant, the DO levels in the CPF treatments were initially higher than in the F and M treatments, but became lower than in the F and M treatments towards the end of the experiment (Fig. 1). This indicates a lower respiration (≈ lower stress) initially by the fish exposed to CPF but higher respiration (≈ higher stress) later compared to the fish exposed to F and M.

**Fig. 2:**
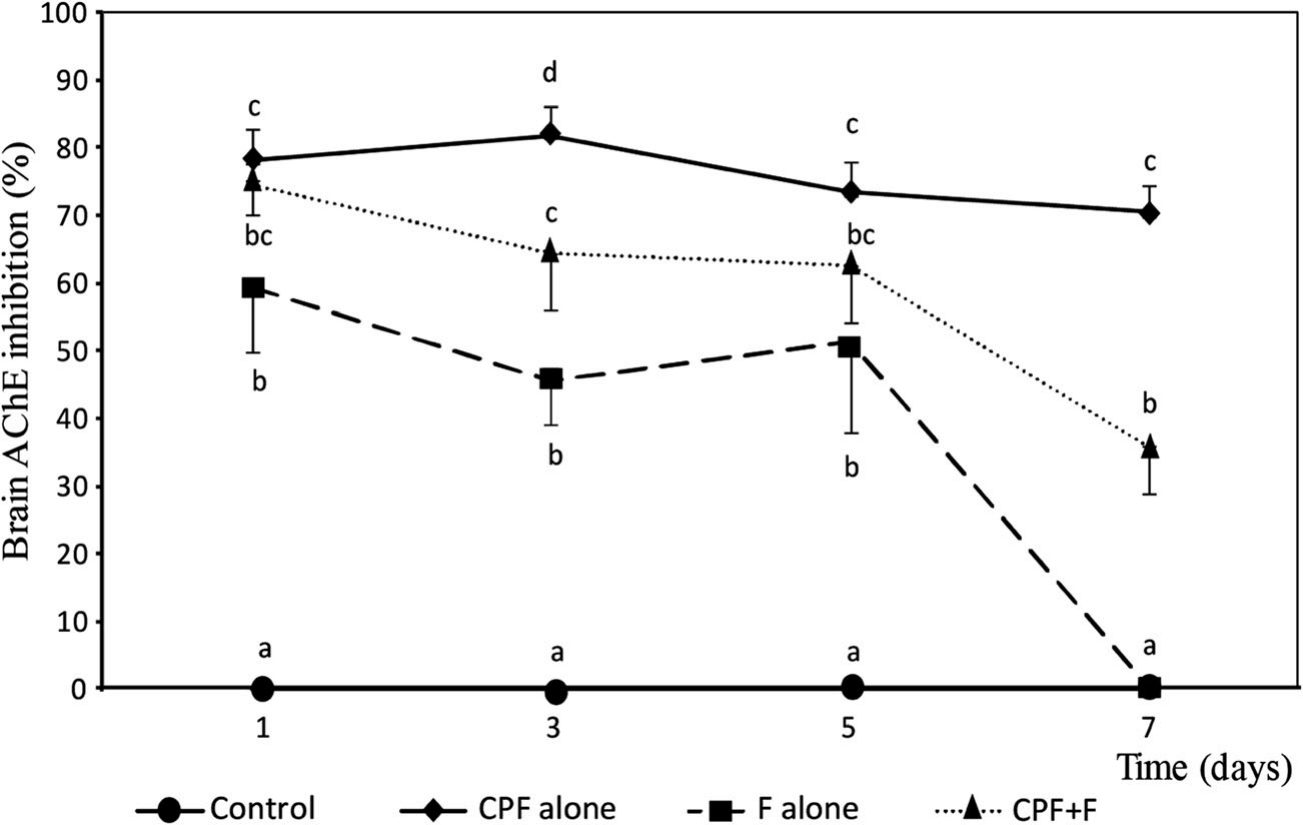
Inhibition (%) of brain acetylcholinesterase activity in climbing perch fingerlings exposed to chlorpyrifos ethyl (♦-CPF alone), fenobucarb (■-F alone), mixture (▲-CPF + F), and control (●). The vertical bars show the standard deviation. Different letters show that means are significantly different at the same sampling time (*P* < 0.05)

One day after the exposure to the insecticides, the brain AChE activity in fish from all treatments decreased significantly as compared to the AChE activity in unexposed fish (*P* < 0.05) (Fig. 2). The inhibition level was highest in fish exposed to CPF (78.2%). This was significantly higher than the inhibition level in fish exposed to F (*P* < 0.05), while there were no significant differences compared with the fish exposed to M (*P* > 0.05) (Fig. 2).

At day 3, the inhibition levels in fish from all treatments were significantly different (*P* < 0.05). The inhibition level in fish exposed to CPF had increased to 81.6%, while the inhibition levels in fish exposed to M and F had decreased to 64.4 and 45.7%, respectively (Fig. 2).

At day 5, the inhibition levels in fish exposed to CPF and M had decreased to 73.4 and 62.7%, respectively, while the inhibition level in fish exposed to F had increased slightly to 51.3% (Fig. 2).

After 7 days, the fish exposed to F had recovered and had the same AChE activity as the fish in the control. The fish exposed to M showed some signs of recovery, but the inhibition level remained above 30% and was significantly higher than the inhibition levels in the fish exposed to F and in the fish from the control (Fig. 1). The fish exposed to CPF showed almost no signs of recovery after 7 days, and the AChE inhibition level (70.7%) was significantly higher than in the fish from the other treatments (*P* < 0.05).

**Fig. 3:**
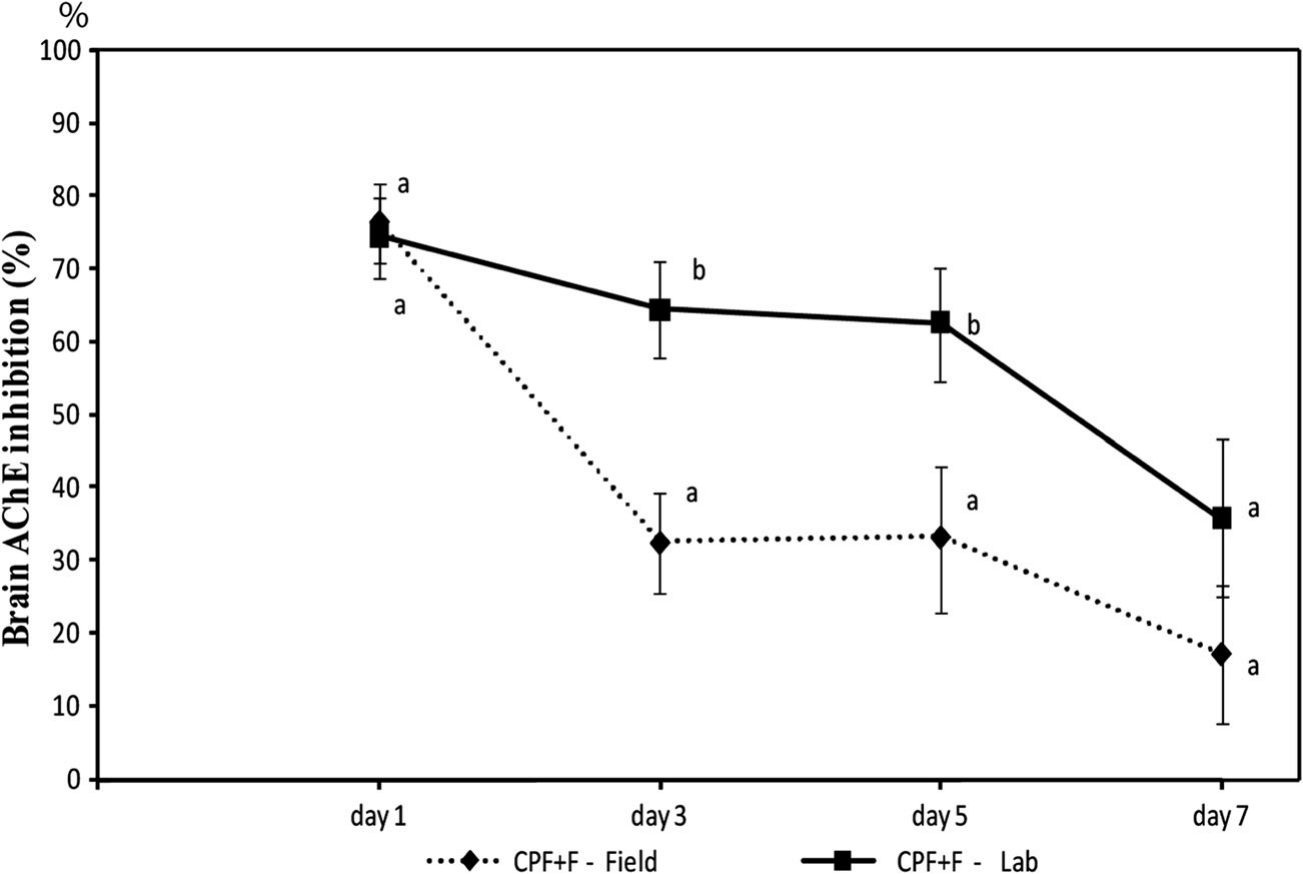
Inhibition of the brain acetylcholinesterase (AChE) activity (% compared to control) in climbing perch fingerlings exposed to mixture of CPF (0.173 mg/L) and F (1.137 mg/L) in the above laboratory experiment (■ CPF + F-Lab) and in climbing perch fingerlings from rice fields sprayed with a mixture of 0.8 L Vitashield 40EC/ha (CPF) and 1.5 L Bassa 50EC/ha (F) (♦ CPF + F-Field) (Tam et al. 2016b). The vertical bars show standard error. Different letters show that means are significantly different at the same sampling time (*P* < 0.05)

The result from the laboratory experiment showed a similar response in fish, as found in a previous study on fish exposed to a mixture of 0.8 L Vitashield 40EC/ha (CPF) and 1.5 L Bassa 50EC/ha (F) in rice fields (Tam et al. 2016b) (Fig. 3). However, the inhibition levels in fish from the laboratory, at day 3 and day 5, were significantly higher than in the fish from the rice fields (*P* < 0.05). At day 7, the inhibition levels in the fish from the laboratory were still above 30%, while the inhibition levels in the fish from the field were reduced to 20% compared to the control.

## Discussion

Despite the importance of climbing perch and other native fish species to local people’s livelihoods in the Mekong Delta, there is little information on the negative effects from agrochemicals on fish and other aquatic organism in the delta (Stadlinger et al. 2016). This lack of information is of major concern, as the ongoing intensification of agriculture must take into account the potential negative effects by pesticides on the regions fisheries resources, including the fast growing aquaculture industry, to avoid unwanted tradeoffs in the overall yield and quality of agriculture and aquaculture products from the delta (Berg et al. 2016; Berg and Tam 2017)*.*

This study shows that commonly used insecticides, such as Vitashield 40EC (CPF) and Bassa 50EC (F), applied separately and in mixtures, caused a significant inhibition of the brain AChE activity in climbing perch fingerlings. Although the concentrations did not cause any direct mortalities among the exposed fish, they were associated with sub-lethal changes in the physiology and behavior that may impair the survival, development, and reproduction of the fish (Kegley et al. 1999). This is of a special concern in the Mekong Delta because farmers often run three crops and spray their fields several times per crop, implying that aquatic organisms in the rice fields may continuously be exposed to and stressed by, elevated levels of pesticides in the water (cf. Stadlinger et al. 2016). The lower oxygen levels in the water with the exposed fish indicate that the fish used an increased amount of energy (increased respiration) to counteract the negative impacts from insecticides, implying that less energy was used for growth. Arunachalam and Palanichamy (1982) proposed that reduced growth is a sensitive indication of chronic toxicity to CPF. Tam et al. (2015a) also found a reduced growth in climbing perch exposed to CPF in rice fields in the Mekong Delta. Hegazi et al. (1898) found that high levels of CPF decreased not only the brain AChE activity but also the glycogen and blood glucose levels in the muscles and liver in *Clarias lazera*. An earlier field study showed that CPF at concentration below 10% of the concentration used in this study (below the detection limit of < 0.03 μg/L) caused 70% inhibition of the brain AChE activity and was associated with both reduced growth and survival rates in climbing perch in rice fields in the Mekong Delta (Tam et al. 2015a). The reasons why no mortalities were found in this study could be due to a lower stress under the more controlled laboratory conditions, and that the experiment period was not long enough to capture these delayed effects.

Other signs of sub-lethal effects found during the experiment included lowered activity, erratic swimming, and rapid grill movements, which is similar to many earlier reported behavior changes of the exposed fish, such as reduced feeding capability, swimming activity, avoidance of predators, and spatial orientation (Pan and Dutta 1998; Sancho et al. 2000; Richendrfer et al. 2012).

The results from this study also indicate that the recovery of the brain AChE activity is quicker in fish exposed to only F than when exposed to a mixture of CPF and F or only CPF. An explanation to this could be that carbamates, such as F, inhibit AChE reversibly (Assis et al. 2011), while the inhibition by CPF often is stronger and more irreversible (Assis et al. 2011). Tam et al. (2016b) found that under field conditions, climbing perch fingerling showed full recovery of the AChE activity 14 days after exposure to a mixture of CPF and F, while the inhibition of the brain AChE activity in climbing perch fingerlings exposed to a similar concentration of only CPF was still above 50% after 12 days with no clear signs of recovery (Tam et al. 2015a). The recovery of the AChE activity in fish is probably because the amount of bioavailable CPF and F in water decreases with time and due to physiology responses by the fish.

Also, the oxygen levels in the different treatments indicate that the fish exposed to only CPF were more stressed (higher respiration compared to the other treatments and the control), at the end of the experiment (day 5 and 7), but were less stressed compared to the other treatments during the first days of the experiment. The comparatively lower initial increase in the respiration by fish treated with only CPF could be because CPF must be activated to form an oxon metabolites via desulfurization reactions to become a potent cholinesterase inhibitor (Chambers and Carr 2002). A recent field study, where climbing perch fingerlings were first exposed to F and later to CPF, confirms that the inhibition of the brain AChE activity by F is quicker but shorter than the effect from CPF (Tam et al. 2016a).

Thus, the combined long-term toxicity effect (> 1 day) by a mixture of CPF and F seems to be lower than the effect from only CPF, because of a less prolonged inhibition of the AChE brain activity by the mixture. This reduced effect is contradictory to several previous studies, which found synergistic effects from the combined exposure to organophosphates and carbamates.

Chen et al. (2014) found that the mixture of CPF and F had a synergistic toxic effect on the AChE activity in common carp. The toxicity of CPF mixed with diazinon appeared to be greater than these chemicals additive toxicity (Bailey et al. 1997; CDFG 1998, 1999a, 1999b). A recent study by Zhao et al. (2010) on *Danio rerio* showed that the toxicity of F mixed with CPF and fipronil was synergistic. Mehler et al. (2007) found that CPF and atrazine had synergistic effects on *Pimephales promelas* and *Chironomus tentans*, while there was no significant difference in the effect on *Lepomis macrochirus* compared to the effect from CPF alone. Several studies on the joint toxicity of CPF and atrazine indicate a synergistic effect on several aquatic species (Pape-Lindstrom and Lydy 1997; Belden and Lydy 2000; Anderson and Lydy 2002; Jin-Clark et al. 2002; Lydy and Linck 2003; Schuler et al. 2005). However, Wacksman et al. (2006) found no synergistic effects, but a small antagonistic effect between atrazine and CPF on the toxicity to *P. promelas*.

The reduced long-term effect from a mixture of CPF and F compared to only CPF, found in this study, could be due to the different bindings of F and CPF to the AChE enzyme. Although both CPF and F inhibit the AChE enzyme, their modes of actions are different. CPF tend to bind stronger to the enzyme because it contains a thiol group (P=S), while the binding to the enzyme of F is less strong and more reversible. Chambers and Carr (2002) noted that phosphorylated AChE is much more stable than the carbamylated AChE and that the spontaneous reactivation is much slower for phosphorylated AChE.

F can also bind directly to AChE enzyme, while CPF need to be activated before it can bind to AChE enzyme (Chambers and Carr 2002). When exposed to a mixture of CPF and F, the majority of the AChE enzymes may therefore initially be blocked by F, which was applied in a higher concentration than CPF, and is more water soluble than CPF. However, with time, the inhibition by F disappears (c.f Fig. 2), because of its reversible bindings. When this happens, the concentrations of both F and CPF in the water have probably decreased to a level where no significant amount of new bindings to the AChE enzyme can be established.

Under field conditions, fish exposed to a mixture of F and CPF recovered even more quickly (c.f. Fig. 3), which probably is because of a larger proportion of the applied CPF, as compared to F, binds to organic matter and the sediments because of CPF’s higher K_oc_ value, and thus cannot bind to the enzyme (Tam 2016).

Thus, it is possible that a combination of F and CPF could have an additive toxic effect initially (< 1 day), but later shows a reduced effect, because of some kind of interference on each other’s mode of action.

## Conclusion

This study shows that the applications of organophosphate and carbamate insecticides, such as CPF and F, impact negatively on native fish species, such as climbing perch. CPF was found to cause a significant and more prolonged inhibition on the brain AChE activity in climbing perch than F. The inhibition by the mixture of CPF and F was significantly higher than the inhibition by only F, but less prolonged and significant lower than the inhibition by only CPF over time.

Still, CPF, F, and the mixture of CPF and F applied at concentrations similar to those applied by rice farmers in the Mekong Delta resulted in a significant reduction in the AChE activity in climbing perch. A recent study by Stadlinger et al. (2016) shows that in the Mekong Delta, most OP and CM compounds are used in concentrations that are not likely to cause any acute toxic effects on aquatic organisms, but may still generate long-term lethal and sub-lethal effects. The results indicate that these effects could be one of the causes of the decline of wild fish populations in rice fields in the Mekong Delta (Anh et al. 2003), and efforts should be made to find safer ways to use pesticides in the Mekong Delta to avoid negative side effects on the environment and peoples’ health.
